# Integrative Methylome and Transcriptome Analysis of DNA Methylation and Sex-Biased Gene Expression in Northern Pike (*Esox lucius*)

**DOI:** 10.3390/ani16182920

**Published:** 2026-09-17

**Authors:** Junjie Zhang, Zhelan Wang, Qian Xiao, Xinan Fu, Sitong Li, Shuhan Chen, Yang Cao, Jiaqing Xu, Xuefei Zhao, Yu Zhang

**Affiliations:** 1 College of Life Sciences, Xinjiang Agricultural University, Urumqi 830052, China; m15286133601@163.com (Z.W.); qianxiao181@163.com (Q.X.); 18087035850@163.com (X.F.); 18160510177@163.com (S.L.); 19335949722@163.com (S.C.); 13116076571@163.com (Y.C.); x320243911@163.com (J.X.); zhaoxuefei147741@163.com (X.Z.); zy107@xjau.edu.cn (Y.Z.); 2 Xinjiang Key Laboratory for Ecological Adaptation and Evolution of Extreme Environment Organisms, Xinjiang Agricultural University, Urumqi 830052, China; 3 Xinjiang Characteristic Aquaculture Research Center, Xinjiang Agricultural University, Urumqi 830052, China; 4 Modern Fisheries Industry College, Xinjiang Agricultural University, Urumqi 830052, China

**Keywords:** northern pike (*Esox lucius*), DNA methylation, sex differentiation, sexual growth dimorphism, integrative transcriptomic and methylomic analysis

## Abstract

Northern pike (*Esox lucius*) exhibits significant sexual growth dimorphism, yet the molecular basis of this sex-biased trait remains largely unexplored. In this study, we performed an integrative analysis of whole-genome DNA methylation (WGBS) and whole-transcriptome (RNA-seq) data from female and male gonadal tissues of *E. lucius*. Promoter methylation exhibited sexually dimorphic associations with gene expression: in females, promoter hypermethylation showed a positive correlation with elevated expression, whereas no such association was observed in males; gene body methylation was not correlated with expression in either sex. Integrative analysis identified a total of 508 methylation-associated differentially expressed genes (mDEGs), comprising 377 Hyper-Down (hypermethylated with downregulated expression) and 131 Hypo-Up (hypomethylated with upregulated expression) genes, from which 13 candidate genes were prioritized. Pathway enrichment analyses implicated TGF-β signaling, Wnt signaling, and progesterone-mediated oocyte maturation pathways, suggesting crosstalk between sex differentiation and metabolic regulation/signal transduction. Collectively, this study provides an exploratory perspective on the epigenetic landscape associated with sex differentiation in *E. lucius.* Our results reveal associations between promoter methylation and the expression of core sex-related genes, including *dmrt1* (male-biased) and *rspo1* (female-biased), which may be relevant to sex differentiation. The potential link between these epigenetic patterns and sexual growth dimorphism remains speculative and requires direct validation using growth-related phenotypic data and tissues.

## Simple Summary

Northern pike is an important fish species in aquaculture, with females growing significantly larger and faster than males. Understanding why females grow bigger could help farmers produce more female fish for market. In this study, we examined both the genetic activity (which genes are turned on or off) and the chemical modifications to DNA that control gene activity in female and male northern pike. We found that these DNA modifications, called methylation, work differently between the sexes. In females, certain regions of DNA that control gene activity were linked to higher gene expression, while this pattern was not seen in males. We identified 508 genes whose activity was associated with these DNA modifications, including 13 key candidate genes. Several important biological pathways involved in sex development were affected by these modifications. Our findings indicate that DNA methylation shows patterns that are linked to sex differentiation, and these patterns may also relate to how fast the fish grows, though this potential link to growth requires further investigation using growth-related data. This knowledge could eventually help aquaculture farmers develop strategies to raise more female northern pike, which grow larger and are more valuable for food production.

## 1. Introduction

Sex determination and differentiation represent one of the most fundamental developmental events in biology, playing critical roles in species’ reproductive strategies, population dynamics, and individual growth, with their regulatory networks governed by both genetic and epigenetic factors [1]. In teleost fishes, the exceptionally diverse sex determination systems, encompassing XX/XY and ZZ/ZW genetic sex determination, temperature-dependent sex determination, as well as genotype–environment interaction models, provide an unparalleled system for investigating the evolution and plasticity of vertebrate sex differentiation mechanisms [2,3]. The direction of sex differentiation in fish is modulated by complex interactions among genetic cascades, hormonal signaling, and epigenetic modifications [4,5]. Consequently, elucidating the molecular underpinnings of sex differentiation is not only of paramount importance for developmental and evolutionary biology but also holds significant implications for aquaculture practices, given that many economically important fish species exhibit pronounced sexual growth dimorphism [6].

Epigenetics refers to heritable phenomena that regulate gene expression through chemical modifications or alterations in chromatin structure without changing the underlying DNA sequence, primarily encompassing DNA methylation, histone modifications, non-coding RNA regulation, and chromatin remodeling [7]. In vertebrates, epigenetic regulation participates throughout the entire process of sex determination and gonadal differentiation. For instance, DNA methylation can influence the transcriptional activity of sex-related genes (such as *dmrt1*, *foxl2*, and *amh*) through changes in the methylation status of promoter CpG islands. In the half-smooth tongue sole (*Cynoglossus semilaevis*), the *dmrt1* promoter exhibits low methylation in the gonads of males and neomales, while it is highly methylated in females [8]. In Nile tilapia (*Oreochromis niloticus*), the methylation level of the *cyp19a1a* promoter region is significantly elevated during high temperature-induced sex reversal of XX individuals [9]. Furthermore, non-coding RNAs, including microRNAs (miRNAs), long non-coding RNAs (lncRNAs), and circular RNAs (circRNAs), can directly target sex-determining key genes or regulate the expression of epigenetic modifier enzymes, thereby forming complex regulatory networks. Numerous studies have reported abundant sexually differentially expressed miRNAs in the gonads of rainbow trout (*Oncorhynchus mykiss*), Nile tilapia, and common carp (*Cyprinus carpio*) [10,11,12]. In the Chinese soft-shell turtle (*Pelodiscus sinensis*), thousands of lncRNAs associated with gonadal differentiation have been identified [13]. Additionally, circRNAs in zebrafish (*Danio rerio*) have been shown to function as miRNA molecular sponges, thereby modulating gene expression [14]. Collectively, these findings underscore the intricate and multifaceted roles of epigenetic mechanisms in regulating sex determination and gonadal differentiation across vertebrates.

Northern pike (*Esox lucius*) is distributed across freshwater basins in North America and northern Eurasia; within China, it primarily inhabits the Irtysh River in the Xinjiang Uygur Autonomous Region, where it is recognized as one of the most economically valuable indigenous fish species [15,16]. This species exhibits pronounced sexual growth dimorphism, with females growing significantly faster than males [17], which makes elucidating the mechanisms underlying sex differentiation particularly relevant for achieving sex-controlled breeding. Regarding the sex determination mechanism, the *amhby* gene has been identified as the master sex-determining gene in northern pike [18]. Further studies have demonstrated that loss of function of the *amhrII* gene leads to male-to-female sex reversal, establishing that *AmhrII* is essential for male sexual development in this species [19]. However, despite the identification of the primary sex-determining gene, the downstream epigenetic regulatory network remains largely unexplored.

We previously employed whole-genome bisulfite sequencing (WGBS) to analyze the DNA methylation patterns in gonadal tissues of female, male, and neomale northern pike. This study generated a total of 361.48 Gb of high-quality reads and identified 66,581 differentially methylated CG regions, 1215 differentially methylated CHG regions, and 3185 differentially methylated CHH regions among the female, male, and neomale groups. Four key differentially methylated candidate genes (*rspo1*, *hsd11b2*, *CYP27A1*, and *smad3*) associated with sex differentiation, as well as sex differentiation-related metabolic pathways including Notch signaling, Wnt signaling, and ovarian steroidogenesis, were screened out [20]. Concurrently, we also completed whole-transcriptome sequencing of female and male gonadal tissues in northern pike, establishing the first whole-transcriptome dataset of female and male gonads for this species. Key sex-biased genes and core pathways involved in sex differentiation were identified, and a dre-miR-107b-centered ceRNA network, along with multiple key sex-biased genes (*FANCL*, *DDX5*, *SRSF5B*, *STAR*, *FDX1B*, *ITGA2B*), were determined as core molecular participants in sex differentiation in this species [21].

Although the above two studies have preliminarily revealed the molecular characteristics of sex differences in northern pike from the methylomic and transcriptomic perspectives, respectively, the regulatory associations between these two omics layers have not yet been systematically elucidated. Here, integration of these two datasets provides two specific advances: at the gene level, it links differential methylation to differential expression to identify methylation-associated DEGs (mDEGs); at the pathway level, it reveals coordinated changes between epigenetic and transcriptional mechanisms in sex-related signaling pathways. These findings extend beyond separate omics descriptions to generate testable hypotheses about methylation–expression associations. Therefore, the present study aims to systematically investigate the associations between DNA methylation and gene transcription in male and female northern pike through the integrative analysis of whole-genome methylation and whole-transcriptome data.

## 2. Materials and Methods

### 2.1. Data Sources

The methylome and transcriptome data utilized in this study were derived from our previous independent investigations. The WGBS data were obtained from Wang et al. (2025) [20], who performed whole-genome bisulfite sequencing on gonadal tissues of female (XX), male (XY), and neomale (XY) northern pike. The WGBS and RNA-seq data were generated from the same individual fish, with gonadal tissues from each biological replicate split for parallel library preparation; both datasets comprised three biological replicates per sex (XX and XY), with no technical replicates, and detailed sequencing statistics are available in the original publications. All fish were 10 months of age, sexually immature, with body length of 37–43 cm and body weight of 478–503 g. Phenotypic sex was determined by histological sectioning and confirmed by PCR-based genotyping. All animal procedures for the original sample collection and sequencing experiments were conducted under the approval of the Animal Welfare and Ethics Committee of Xinjiang Agricultural University (protocol code 2022027). The present integrative analysis is a secondary analysis of previously generated datasets and did not require additional animal ethics approval. The RNA-seq data were obtained from Zhang et al. (2026) [21], who conducted whole-transcriptome sequencing on gonadal tissues of female and male northern pike. Gene annotation was based on NCBI RefSeq release 104 (GTF format). The procedures for quality control, read alignment, methylation calling, differentially methylated region (DMR) identification, and differentially expressed gene (DEG) identification are described in detail in the aforementioned publications.

Promoter regions were defined as 2000 bp upstream to 200 bp downstream of the transcription start site (TSS). For methylation analysis, only CpG sites with a minimum coverage of 10× were retained. DMRs were identified using the DSS package (version 2.12.0; https://bioconductor.org/packages/release/bioc/html/DSS.html, accessed on 4 June 2025); DEGs were identified using DESeq2 (version 1.34.0; https://bioconductor.org/packages/release/bioc/html/DESeq2.html, accessed on 4 June 2025) or edgeR (version 3.32.1; https://bioconductor.org/packages/release/bioc/html/edgeR.html, accessed on 4 June 2025). Lowly expressed genes (CPM < 1) were filtered prior to DEG analysis. All *p*-values were adjusted using the Benjamini–Hochberg FDR method.

For DMR identification, we applied the same thresholds as previously established for this species: |Δβ| ≥ 0.2 and FDR < 0.05, with each DMR containing a minimum of 5 CpG sites. For DEG identification, the significance threshold was set at FDR < 0.05 with an absolute |log_2_(fold change)| ≥ 1. All statistical thresholds were uniformly applied across all comparative groups. The WGBS data comprised three biological replicates per sex, and the RNA-seq data comprised three biological replicates per sex.

Only the female (XX) vs. male (XY) comparison was used in this integrative study; neomale data were excluded as they represent a sex-reversed condition that warrants separate investigation.

### 2.2. Association Analysis Between Methylation and Expression Distribution

To evaluate the impact of methylation levels on gene expression distribution, genes in female and male fish were separately divided into five equal groups (group.1st to group.5th) based on their promoter (defined as the genomic region from 2000 bp upstream to 200 bp downstream of the transcription start site, TSS) and gene body CG methylation levels (i.e., the average methylation percentage of all CpG sites within each region), ranging from low to high. The differences in gene expression distribution were then compared among these groups. To quantitatively assess the genome-wide association between promoter methylation and gene expression, Spearman’s rank correlation coefficient (ρ) was calculated for each gene using all six samples (three females and three males). The distribution of ρ values across all genes was summarized. Additionally, genes were dichotomized into high- and low-methylation groups based on the median promoter methylation level, and expression differences between the two groups were evaluated using the Mann–Whitney U test.

### 2.3. Four-Quadrant Association Analysis Between Methylation and Expression, and mDEGs Screening

Genes were considered as ‘DMR-annotated genes’ if they contained at least one DMR within their promoter region (as defined in Section 2.2) or gene body. The four-quadrant classification was applied only to the intersection of DMR-annotated genes and DEGs that had already passed the stringent thresholds described in Section 2.1 for both methylation and expression. To systematically dissect the directional associations between methylation differences and expression differences, the intersection genes of DMR-annotated genes and DEGs were classified into four categories according to the direction of methylation difference and expression difference, following the four-quadrant classification method established by Wang et al. (2025) [20] in their integrative analysis of sexual dimorphism in fish: Hyper-Up (methylation log_2_FC > 0, expression log_2_FC > 0), Hyper-Down (methylation log_2_FC > 0, expression log_2_FC < 0), Hypo-Up (methylation log_2_FC < 0, expression log_2_FC > 0), and Hypo-Down (methylation log_2_FC < 0, expression log_2_FC < 0). Genes classified as Hyper-Down and Hypo-Up in the promoter region were selected as “methylation-associated differentially expressed genes (mDEGs).” To quantify the degree of inverse regulation between methylation and expression, the Relative Score (RS) was defined as |methylation log_2_FC| × |expression log_2_FC|. The top 10 mDEGs ranked by descending RS values were selected as core candidate genes linked to promoter methylation.

### 2.4. Integrated KEGG/GO Enrichment Pathway Analysis

GO and KEGG enrichment analyses were performed using all protein-coding genes annotated in the *E. lucius* reference genome (GCF_011004845.1) as the background gene set. Gene Ontology (GO) term enrichment analysis was performed using the R package GOseq(version 1.44.0; https://bioconductor.org/packages/release/bioc/html/goseq.html, accessed on 4 June 2025), with a significance threshold of adjusted *p* < 0.05. Kyoto Encyclopedia of Genes and Genomes (KEGG) pathway enrichment analysis was performed using KOBAS software (version 3.0; http://kobas.cbi.pku.edu.cn, accessed on 4 June 2025). All *p*-values were adjusted using the Benjamini–Hochberg FDR method.

To identify signaling pathways showing coordinated involvement of both transcriptional and epigenetic changes, the intersection of DEGs and promoter DMGs (Differentially Methylated Genes) was first obtained to generate a “methylation–expression overlapping gene set,” which was then subjected to GO functional annotation enrichment analysis and KEGG pathway enrichment analysis. Subsequently, independent KEGG pathway enrichment analyses were performed separately on all DEGs and all promoter DMGs, and the enrichment results of the two sets were cross-compared at the pathway level. Pathways meeting either of the following criteria were defined as “core epigenetic, transcriptional synergistic pathways”: (a) the pathway reached statistical significance (FDR < 0.05) in both DEG enrichment and promoter DMG enrichment; or (b) the pathway did not reach the significant enrichment threshold in one omics layer, but multiple key genes within the pathway were simultaneously present in the intersection of DEGs and DMGs. The significance threshold for all enrichment analyses was set at FDR < 0.05.

## 3. Results

### 3.1. Genome-Wide Landscape of Methylation and Transcriptional Expression Correlation

Since CG methylation is the predominant form of methylation in the genomes of teleost fish, this study focused on CG-context methylation in the methylation–transcriptional association analyses. The methylation data for CHG and CHH contexts have been reported in detail by Wang et al. (2025) [20] and are not reiterated here. To evaluate the potential association of promoter DNA methylation and gene transcription, genes in female and male fish were separately divided into five equal groups (group.1st to group.5th) based on their promoter CG methylation levels, ranging from low to high, and the gene expression distributions were compared among these groups.

**Figure 1:**
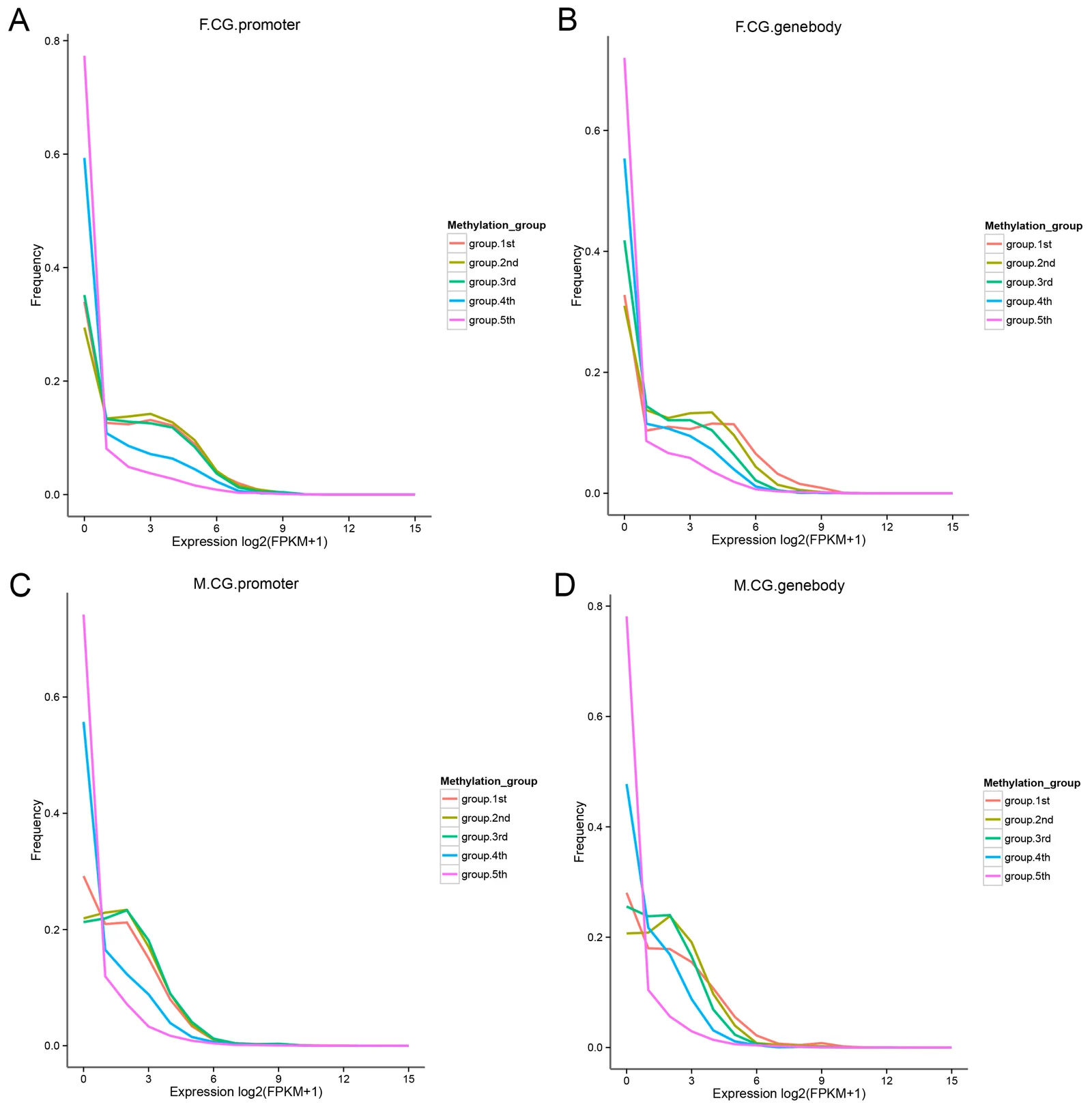
Distribution of gene expression across promoter and gene body methylation levels in females and males. (**A**) Female: promoter methylation groups; (**B**) Female: gene body methylation groups; (**C**) Male: promoter methylation groups; (**D**) Male: gene body methylation groups. Note: Genes were divided into five equal groups (group.1st to group.5th) based on CG methylation levels from low to high. The x-axis represents gene expression levels (log_2_(FPKM+1)), and the y-axis represents the proportion of genes in each expression interval.

In females, group.1st (the lowest methylation group) exhibited the highest proportion of genes in the low-expression interval (log_2_(FPKM+1) < 1) at 0.77, while group.5th (the highest methylation group) showed the lowest proportion at 0.57. Moreover, as methylation levels increased progressively from group.1st to group.5th, the proportion of low-expression genes exhibited a gradual declining trend (Figure 1A). In males, the expression distribution curves of the five groups were highly overlapping, with group.1st and group.5th showing nearly identical proportions in the low-expression interval (0.54 vs. 0.54) (Figure 1C).

Unlike the promoter region, gene-body methylation exhibited a distinct pattern in relation to gene expression. Genes in female and male fish were separately divided into five equal groups based on their gene body CG methylation levels, and the expression distributions were compared among these groups. In both females and males, the five expression distribution curves from group.1st to group.5th were completely overlapping (Figure 1B,D), suggesting that gene body methylation levels were not associated with gene expression distribution regardless of methylation status.

Genome-wide Spearman correlation analysis revealed a strong inverse association between promoter methylation and gene expression (median ρ = −0.76, interquartile range: −0.91 to −0.47), consistent with the substantial epigenetic and transcriptional divergence between male and female gonadal tissues. When genes were stratified into high- and low-methylation groups, the high-methylation group exhibited significantly lower expression than the low-methylation group (Mann–Whitney U test, *p* < 0.001). These findings suggest a robust statistical link between promoter hypermethylation and transcriptional suppression, although causality cannot be inferred from these correlational data.

### 3.2. Screening of Promoter Methylation-Associated Differentially Expressed Genes (mDEGs)

**Figure 2:**
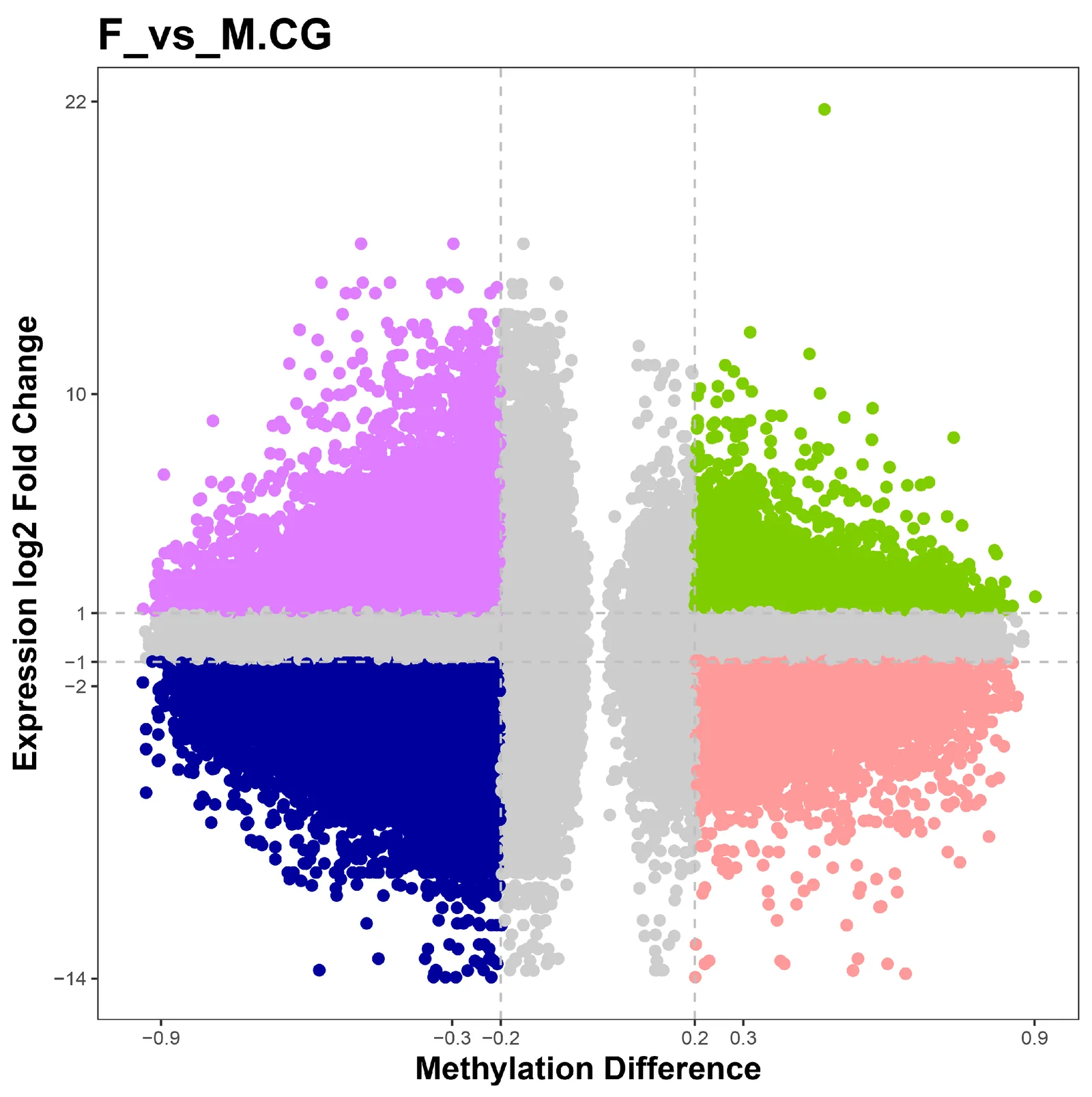
Four-quadrant classification of promoter methylation-associated genes. Note: purple, Hypo-Up (hypomethylated and upregulated); dark blue, Hypo-Down (hypomethylated and downregulated); green, Hyper-Up (hypermethylated and upregulated); pink, Hyper-Down (hypermethylated and downregulated); gray, genes not passing both methylation and expression thresholds and not assigned to any quadrant.

To further identify candidate sex-biased genes putatively associated with promoter methylation, we performed an intersection analysis between genes containing DMRs in the promoter region and DEGs, and classified them using a four-quadrant approach based on the directions of methylation differences and expression differences (Figure 2). Among the genes with both methylation and expression differences in the promoter region, two negatively correlated patterns, Hyper-Down (hypermethylated with downregulated expression) and Hypo-Up (hypomethylated with upregulated expression), comprised 377 and 131 genes, respectively. Since the directions of methylation differences and expression differences were opposite for these genes, their expression changes were considered as potentially linked to promoter DNA methylation. These two categories, totaling 508 genes, were defined as “methylation-associated differentially expressed genes (mDEGs).”

Table 1 lists the top 10 mDEGs significantly associated with promoter methylation, including their methylation fold changes, expression fold changes, and Relative Scores. Among them, *hoxd3a*, *hoxa3a*, *gata2a*, *LOC105007941*, *mab21l2*, *LOC105013737*, *hoxb3a*, *hoxd4a*, *LOC114828856,* and *tppp3* all exhibited inverse methylation–expression association patterns between methylation and expression, with *LOC105007941* and *hoxd4a* showing the strongest inverse methylation–expression changes (highest RS values).

### 3.3. Integrated Enrichment Pathway Analysis Identifies Signaling Pathways Associated with Sex Differentiation

**Figure 3:**
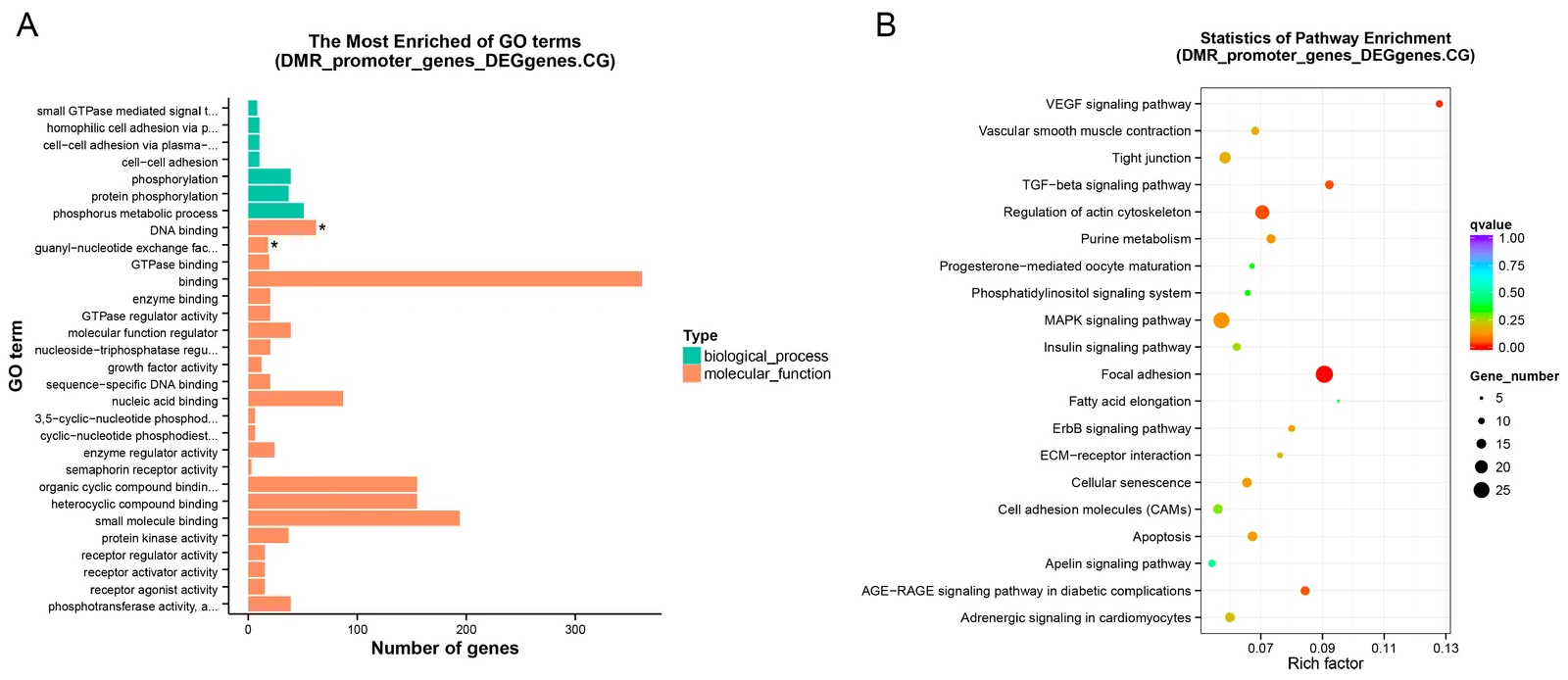
Functional annotation and pathway enrichment of mDEGs. (**A**) GO functional enrichment analysis. (**B**) KEGG pathway enrichment analysis. The asterisk (*) indicates statistical significance (FDR < 0.05). In (A), the truncated GO term labels correspond, from top to bottom, to the complete GO terms listed in Supplementary Table S1.

These pathways showed coordinated changes at both transcriptional and epigenetic levels; we performed GO functional annotation enrichment analysis and KEGG pathway enrichment analysis on the mDEGs (Figure 3).

KEGG pathway enrichment analysis revealed that the mDEGs were significantly enriched in multiple signaling pathways closely associated with sex differentiation and gonadal development (Figure 3B), including the TGF-β signaling pathway, MAPK signaling pathway, progesterone-mediated oocyte maturation, and apoptosis (q < 0.05). GO functional enrichment analysis showed that the mDEGs were predominantly enriched in biological processes and molecular functions such as cell–cell adhesion via plasma membrane, protein phosphorylation, DNA binding, growth factor activity, and sequence-specific DNA binding (*p* < 0.05) (Figure 3A).

### 3.4. Epigenetic/Transcriptional Associations of Core Sex-Biased Genes

Among the mDEGs, a total of four candidate genes associated with sex differentiation were identified. Of these, *dmrt1* and *rspo1*, serving as representative genes of the core pathways for testicular and ovarian differentiation, respectively, exhibited pronounced inverse methylation–expression association patterns, with Relative Scores of 0.52 and 0.47, respectively. Therefore, these two genes were selected as examples to further dissect the modes of association between promoter methylation on key sex differentiation genes.

**Figure 4:**
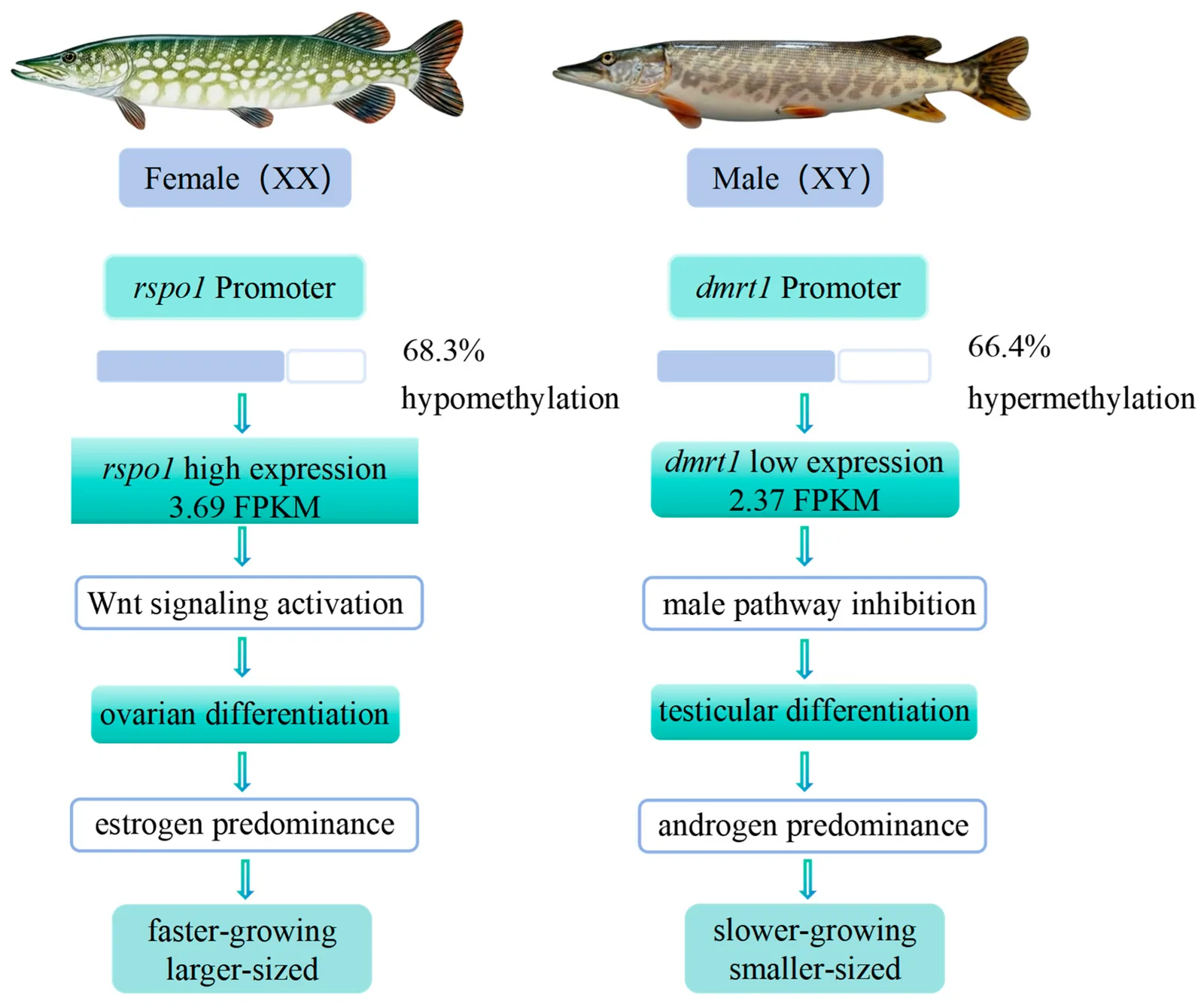
Schematic overview of DNA methylation patterns associated with sex differentiation and the speculative link to growth dimorphism in *E. lucius*, illustrating opposite correlative patterns for the male-biased gene *dmrt1* and female-biased gene *rspo1*.

Pairwise analysis revealed that the promoter methylation and gene expression of both genes exhibited inverse relationships, yet in completely opposite directions (Figure 4): dmrt1 displayed a hypomethylation–high expression pattern in males (methylation: 49.9% in males vs. 66.4% in females; expression: 5.75 vs. 2.37 FPKM in males vs. females), whereas *rspo1* displayed a hypomethylation–high expression pattern in females (methylation: 68.3% in females vs. 96.5% in males; expression: 3.69 vs. 1.92 FPKM in females vs. males). These results indicate that promoter hypomethylation represents a common epigenetic feature associated with high expression of sex-biased key genes in their respective sexes. Notably, the inverse methylation–expression directions of the two genes were completely opposite, consistent with a model of opposite methylation–expression patterns in males and females.

## 4. Discussion

### 4.1. Discussion of the Genome-Wide Landscape of Methylation and Transcriptional Expression Correlation

In this study, genes were grouped by promoter methylation levels to compare gene expression distributions across groups, revealing prominent sex-specific differences in the promoter methylation-expression relationship between female and male gonads. In females, promoter methylation showed a positive correlation trend with gene expression: the high-methylation group (group.5th) had the lowest proportion of low-expression genes (0.57), whereas the low-methylation group (group.1st) displayed the highest proportion (0.77). This observation is not entirely consistent with the classical model of “promoter methylation represses gene expression,” but it is not without precedent in fish. In an integrative analysis of the gonadal methylome and transcriptome in the Senegalese sole (*Solea senegalensis*), DNA methylation has been reported to have either repressive or enhancing effects on gene expression depending on the genomic context, suggesting that the methylation–expression relationship may vary across species, tissues, and genomic regions [22]. Positive methylation-expression correlations have also been reported for subsets of genes in *Scatophagus argus* [23]. In addition, work on zebrafish embryos indicates that promoter hypomethylation is necessary but not sufficient for gene activation [24].

This positive correlation in females deviates from the classical model of methylation-mediated repression. Several non-mutually exclusive explanations exist, including genomic context and CpG density, transcription factor occupancy and chromatin state, gene category (e.g., housekeeping vs. tissue-specific), and reverse causality, as active transcription itself may influence methylation patterns. Our data cannot distinguish among these possibilities; therefore, this association should be interpreted as correlational rather than causal.

In males, by contrast, expression distributions across the five methylation strata largely overlapped (group.1st vs. group.5th: 0.54 vs. 0.54), pointing to negligible associations of promoter methylation with global gene expression patterns. Such sex-specific discrepancies may be associated with divergent transcriptional requirements in ovaries versus testes, in line with sexually dimorphic gonadal methylation profiles described in zebrafish [25]. Ovarian development requires coordinated activation and silencing of large sets of oocyte-related genes within complex networks in which methylation is involved; testicular development, on the other hand, may be more extensively associated with alternative network mechanisms such as transcription-factor-driven circuits.

Unlike the promoter region, gene-body methylation showed no detectable link with gene expression. Expression distribution curves across five gene-body-methylation strata were fully superimposed in both sexes, suggesting that gene expression distribution was independent of gene body methylation status. This finding is consistent with the functional understanding of gene body methylation. Unlike promoter methylation, which is primarily associated with transcription initiation, gene body methylation has been reported to be involved in maintaining transcriptional fidelity (by suppressing aberrant transcription from intragenic cryptic promoters), regulating alternative splicing, and inhibiting transposable element activity, with limited direct effects on transcription levels [26,27,28,29]. In a study of common carp (*Cyprinus carpio*) gonads, although gene body differentially methylated genes were enriched in the Wnt signaling pathway, TGF-β signaling pathway, and GnRH signaling pathway, their methylation changes did not directly correspond to expression changes, further suggesting that gene body methylation may be associated with sex differentiation indirectly [30].

We acknowledge that our grouping-based analysis provides distributional comparisons rather than direct gene-level quantitative correlations. While effective for visualizing genome-wide trends, it does not provide individual gene-level correlation estimates. Future gene-level regression or correlation analyses would provide stronger quantitative evidence.

Taken together, our results highlight marked sex- and region-specific features of DNA-methylation-gene-expression coupling in northern pike gonads. Promoter methylation trended positively with gene expression in females but exhibited no obvious association in males, whereas gene-body methylation was uncoupled from expression in both sexes. Therefore, promoter methylation was associated with transcriptional patterns predominantly in the ovary, and male gonadal gene expression may be more extensively associated with other regulatory layers.

### 4.2. Functional Implications of Promoter Methylation-Associated Differentially Expressed Genes (mDEGs)

The negative association between promoter DNA methylation and gene expression has been reported in multiple fish studies. In the sex reversal study of the half-smooth tongue sole (*Cynoglossus semilaevis*), promoter methylation was confirmed as a key mechanism regulating sex-biased gene expression [8]. In the Senegalese sole (*Solea senegalensis*), DNA methylation was shown to exert either repressive or enhancing effects on gene expression depending on the genomic context [22]. In the present study, through four-quadrant association analysis, we identified 508 mDEGs (377 Hyper-Down and 131 Hypo-Up) from genes exhibiting both methylation and expression differences in the promoter region, further supporting the widespread negative association of promoter DNA methylation in gene transcription, consistent with the aforementioned findings.

Notably, the number of Hyper-Down genes (377) was significantly higher than that of Hypo-Up genes (131). This quantitative disparity may be due to a preferred pattern of methylation modification in the gonads of male and female northern pike, suggesting that promoter hypermethylation-mediated transcriptional repression (Hyper-Down) may be more common than hypomethylation-mediated transcriptional activation (Hypo-Up) during sex differentiation. A similar phenomenon has been reported in the integrative analysis of the gonadal methylome and transcriptome in the spotted scat (*Scatophagus argus*), where hypermethylation of promoter regions in male-biased genes was closely associated with their downregulated expression [23]. This asymmetry may be related to the more prominent requirement for gene silencing during gonadal development-for instance, the repression of female pathway genes such as cyp19a1a in the testis, and the suppression of male pathway genes such as amh in the ovary.

Among the Top 10 mDEGs, multiple genes possess well-defined functions in sex differentiation or developmental processes. *gata2a* (GATA-binding protein 2a), a member of the GATA transcription factor family, has been reported to be involved in the development of primordial germ cells in zebrafish [31]. *hoxd3a*, *hoxa3a*, and *hoxb3a* belong to the HOX family of transcription factors, which are known to play roles in vertebrate gonadal development and reproductive tract patterning [32,33]. *mab21l2*, a member of the MAB-21 family, is involved in cell fate determination in Caenorhabditis elegans, although the sex differentiation function of its fish homologs remains to be elucidated [34]. *tppp3* (tubulin polymerization-promoting protein 3) is involved in cytoskeletal dynamics regulation [35]. Notably, *LOC105007941*, *LOC105013737*, and *LOC114828856* are predicted genes annotated with LOC identifiers, whose specific functions in northern pike sex differentiation await further investigation. Functional evidence for these genes is currently limited; their promoter methylation suggests potential involvement in sex differentiation.

The identification of these candidate genes provides important clues for further understanding the epigenetic landscape underlying sex differentiation in *E. lucius*. Among them, *mab21l2*, although less frequently reported in fish sex differentiation studies, has known functions in cell fate determination, germ cell development, and mRNA stability regulation, suggesting potential roles in sex differentiation through multiple pathways. Future functional experiments, such as gene knockout/knockdown and methyltransferase inhibitor treatments, are warranted to further investigate the specific functions and methylation-associated features of these candidate genes in northern pike sex differentiation.

### 4.3. Integrated Enrichment Pathway Analysis Suggests Signaling Pathways Associated with Sex Differentiation

KEGG pathway enrichment analysis of promoter-associated mDEGs identified multiple signaling pathways closely associated with sex differentiation and gonadal development, among which the TGF-β signaling pathway, MAPK signaling pathway, and progesterone-mediated oocyte maturation pathway were most significantly enriched. The TGF-β signaling pathway is recognized as one of the core pathways involved in vertebrate testicular differentiation, and its key ligand gene *amh* has been shown to exhibit male-biased expression and be linked to promoter methylation in multiple fish species [36]. In the present study, the significant enrichment of pathway-related genes among mDEGs suggests that promoter methylation is associated with the expression of key TGF-β pathway genes such as *amh,* which may be relevant to testicular differentiation in *E. lucius*. The MAPK signaling pathway plays a critical role in the resumption of oocyte meiosis, while the progesterone signaling pathway is directly involved in the process of oocyte maturation [37]. The significant enrichment of these two pathways among mDEGs further suggests that promoter methylation shows associations with the processes related to female germ cell development.

Notably, the aforementioned pathways were not only significantly enriched among mDEGs but also reached significant levels in independent enrichment analyses of all DEGs. This finding suggests that the TGF-β signaling pathway, MAPK signaling pathway, and progesterone-mediated oocyte maturation pathway show coordinated changes at both the transcriptional and epigenetic levels during sex differentiation in northern pike, as genes in these pathways exhibited both differential expression and differential promoter methylation. These coordinated patterns may be associated with significant differences between males and females in key signaling pathways governing gonadal development, which may be associated with gonadal differentiation proceeding toward different directions.

From an evolutionary conservation perspective, the epigenetic dynamics of the TGF-β signaling pathway may have general significance in fish sex differentiation. In studies of common carp (*Cyprinus carpio*) gonads, differentially methylated genes were also significantly enriched in the TGF-β signaling pathway [30]. In the half-smooth tongue sole (*Cynoglossus semilaevis*), methylation–expression synergistic changes in the cell cycle and Hippo signaling pathways have been reported to be associated with a speculative link to female-biased growth dimorphism [38]. The results of the present study are consistent with these findings, further supporting the conserved nature of epigenetic dynamics in the TGF-β signaling pathway in fish sex differentiation, while also suggesting that the pathways coordinately associated with methylation may exhibit species-specificity across different fish species.

GO functional enrichment analysis further identified the molecular functional characteristics of mDEGs. The mDEGs were significantly enriched in GO terms such as “sequence-specific DNA binding” and “growth factor activity.” Among these, the “sequence-specific DNA binding” term is closely associated with transcription factor regulation, suggesting that promoter methylation may be indirectly associated with the expression of key sex differentiation genes, potentially through effects on the DNA accessibility of transcription factor binding sites. This pattern is consistent with the classical model of epigenetic modification: CpG island methylation in promoter regions has been reported to obstruct transcription factor binding to DNA, thereby being correlated with transcriptional repression [39,40]. The “growth factor activity” term is associated with cell signal transduction [30], further supporting the biological significance of the aforementioned pathway enrichment results.

Pathway enrichment analyses are inherently hypothesis-generating and do not demonstrate functional regulation by DNA methylation. The co-enrichment of DEGs and DMGs in the same pathways suggests potential coordination, but direct causal relationships require experimental validation (e.g., targeted methylation editing or pathway-specific perturbations).

### 4.4. Epigenetic-Transcriptional Associations of Core Sex-Biased Genes

Through the analysis of promoter methylation and expression associations of two representative genes, dmrt1 and *rspo1*, this study further highlights the association of promoter methylation in key sex differentiation genes. The hypomethylation and high-expression pattern of *dmrt1* in males is consistent with its known function as a key transcription factor in testicular differentiation; this pattern is consistent with the hypothesis that promoter demethylation could relieve transcriptional repression, potentially facilitating *dmrt1* transcription in the testis [41]. Conversely, the hypomethylation and high expression pattern of *rspo1* in females aligns with its role in expression changes in the Wnt signaling pathway in ovarian differentiation, where promoter demethylation is associated with activation of Wnt signaling and correlates with ovarian differentiation [42,43,44].

Notably, *dmrt1* and *rspo1* were among the differentially expressed genes significantly enriched in the TGF-β signaling pathway and the Wnt signaling pathway, respectively, both of which were significantly enriched in the KEGG enrichment analysis of mDEGs. Therefore, the methylation patterns of *dmrt1* and *rspo1* are not isolated phenomena but rather represent the overall association of promoter methylation with two core sex differentiation pathways. In males, promoter methylation tends to be associated with repressed expression of female pathway genes (such as *rspo1*) while correlating with elevated expression of male pathway genes (such as *dmrt1*); the opposite pattern is observed in females. This opposite methylation-expression association is consistent with observations in the sex reversal study of the half-smooth tongue sole (*Cynoglossus semilaevis*), where the promoter methylation status of sex-biased genes was inversely associated with their expression direction [8,38], and these epigenetic patterns showed reversible reprogramming during sex reversal.

While the opposite methylation–expression patterns of *dmrt1* and *rspo1* are consistent with a model of opposing regulation, functional experiments, such as CRISPR/dCas9-based targeted methylation editing, promoter methylation validation (e.g., bisulfite sequencing of individual CpG sites), or chromatin accessibility analysis (e.g., ATAC-seq) are required to test this hypothesis and establish a causal switch-like mechanism. The model presented here should therefore be regarded as a working hypothesis rather than a demonstrated mechanism.

It should be noted that the RNA-seq and WGBS data used in this study were generated from bulk gonadal tissues, which represent the average signal across all cell types. Therefore, although we observed sex-biased expression and methylation patterns for genes such as dmrt1 and *rspo1*, we cannot assign these patterns to specific cell populations (e.g., Sertoli cells or granulosa cells). Our conclusions regarding the proposed model are therefore based on tissue-level associations. Male and female gonads differ markedly in cellular composition. Thus, observed methylation/expression differences could partly reflect cell type proportion differences rather than cell-intrinsic regulation—an inherent limitation of bulk tissue analyses. Future studies employing in situ hybridization (ISH), immunohistochemistry (IHC), or single-cell RNA-seq are required to resolve the cell-type-specific localization of these epigenetic and transcriptional changes.

Neomale WGBS and RNA-seq data are available but were not included here; they will be analyzed separately in future studies to investigate sex reversal mechanisms.

In summary, this study presents an epigenetic model for sex differentiation and its speculative link to growth dimorphism in northern pike (Figure 4): in females, demethylation of the *rspo1* promoter is associated with expression changes in the Wnt signaling pathway, which correlates with ovarian differentiation and genes involved in estrogen synthesis, and may be relevant to rapid growth; in males, demethylation of the *dmrt1* promoter is associated with expression changes in the TGF-β signaling pathway, which correlates with testicular differentiation and genes involved in androgen synthesis. Through differential demethylation/hypermethylation of promoter methylation, these two key genes show opposite patterns in association with sex differentiation in males and females, which may contribute to the basis for growth dimorphism, with females growing larger than males. This model summarizes the divergent methylation–expression patterns associated with sex differentiation [45] and provides a preliminary basis for investigating epigenetic correlates of sexual growth dimorphism [46], though the link to growth remains speculative and requires validation using growth-related phenotypic data.

## 5. Conclusions

This integrative analysis illustrates that promoter methylation is differentially associated with sex differentiation in northern pike: hypomethylation of *dmrt1* in males and *rspo1* in females correlates with testicular and ovarian differentiation, respectively, and is associated with sexual growth dimorphism. The identification of coordinated pathways (TGF-β, Wnt) and 508 methylation-associated differentially expressed genes (mDEGs) provides a useful foundation for future studies on sex-controlled breeding in fish. However, the absence of functional validation represents a major limitation. The associations identified here are correlational and require experimental validation to establish causality. The proposed model should therefore be regarded as a working hypothesis rather than a demonstrated mechanism.

## Figures and Tables

**Table 1:** Promoter methylation-associated differentially expressed genes (mDEGs). Note: Relative Score (RS): |methylation log_2_FC| × |expression log_2_FC|. Note: Relative Score (RS) = |methylation log_2_FC| × |expression log_2_FC|. All genes listed meet the significance thresholds for both methylation (|Δβ| ≥ 0.2, FDR < 0.05) and expression (|log_2_FC| ≥ 1, FDR < 0.05).

Gene ID	Gene Name	Type	Methylation log_2_FC	Expression log_2_FC	Relative Score
105016228	hoxd3a	Hyper-Down	5.119957668	−2.251641316	11.52
105023656	hoxa3a	Hyper-Down	5.662607246	−1.967148641	11.15
105017313	gata2a	Hyper-Down	5.286266802	−1.189744906	6.29
105007941	LOC105007941	Hyper-Down	5.01254628	−3.241584239	16.23
105020925	mab21l2	Hyper-Down	5.318508544	−1.692020232	8.99
105013737	LOC105013737	Hyper-Down	5.403318936	−1.267648927	6.86
105013251	hoxb3a	Hyper-Down	5.043714776	−1.560866604	7.86
105016229	hoxd4a	Hyper-Down	5.047605095	−2.775415961	14.04
114828856	LOC114828856	Hypo-Up	−2.214088172	1.854466358	4.09
105025477	tppp3	Hypo-Up	−2.293195965	1.704080341	3.90

## Data Availability

The results of whole-genome methylation sequencing of *Esox lucius* have been deposited in the BioProject database of the National Center for Biotechnology Information (NCBI) with the accession number PRJNA1356644 (WGBS methylome data) and PRJNA1455254 (RNA-seq transcriptome data). The public access URL is: https://www.ncbi.nlm.nih.gov/bioproject/PRJNA1356644 (accessed on 10 December 2025); https://www.ncbi.nlm.nih.gov/bioproject/PRJNA1455254 (accessed on 19 April 2026).

## Supplementary Materials

The following supporting information can be downloaded at: https://www.mdpi.com/article/10.3390/ani16182920/s1.
